# Flexible Layered-Graphene Charge Modulation for Highly Stable Triboelectric Nanogenerator

**DOI:** 10.3390/nano11092276

**Published:** 2021-09-01

**Authors:** Mamina Sahoo, Sz-Nian Lai, Jyh-Ming Wu, Ming-Chung Wu, Chao-Sung Lai

**Affiliations:** 1 Department of Electronic Engineering, Chang Gung University, Guishan District, Taoyuan City 33302, Taiwan; msahoo12@gmail.com; 2 Department of Materials Science and Engineering, National Tsing Hua University, Hsinchu 30010, Taiwan; snlai712@gapp.nthu.edu.tw (S.-N.L.); jmwuyun@gapp.nthu.edu.tw (J.-M.W.); 3 High Entropy Materials Center, National Tsing Hua University, Hsinchu 30010, Taiwan; 4 Department of Chemical and Materials Engineering, Chang Gung University, Taoyuan City 33302, Taiwan; mingchungwu@mail.cgu.edu.tw; 5 Artificial Intelligence and Green Technology Research Center, Chang Gung University, Guishan District, Taoyuan City 33302, Taiwan; 6 Department of Materials Engineering, Ming Chi University of Technology, Taishan District, New Taipei City 24301, Taiwan; 7 Department of Nephrology, Chang Gung Memorial Hospital, Guishan District, Taoyuan City 33305, Taiwan

**Keywords:** graphene, triboelectric nanogenerator, charge trapping layer, flexible, stability, energy harvesting

## Abstract

The continuous quest to enhance the output performance of triboelectric nanogenerators (TENGs) based on the surface charge density of the tribolayer has motivated researchers to harvest mechanical energy efficiently. Most of the previous work focused on the enhancement of negative triboelectric charges. The enhancement of charge density over positive tribolayer has been less investigated. In this work, we developed a layer-by-layer assembled multilayer graphene-based TENG to enhance the charge density by creatively introducing a charge trapping layer (CTL) Al_2_O_3_ in between the positive triboelectric layer and conducting electrode to construct an attractive flexible TENG. Based on the experimental results, the optimized three layers of graphene TENG (3L-Gr-TENG) with CTL showed a 30-fold enhancement in output power compared to its counterpart, 3L-Gr-TENG without CTL. This remarkably enhanced performance can be ascribed to the synergistic effect between the optimized graphene layers with high dielectric CTL. Moreover, the device exhibited outstanding stability after continuous operation of >2000 cycles. Additionally, the device was capable of powering 20 green LEDs and sufficient to power an electronic timer with rectifying circuits. This research provides a new insight to improve the charge density of Gr-TENGs as energy harvesters for next-generation flexible electronics.

## 1. Introduction

With the advent of the fourth industrial revolution, the demand for flexible, portable and wearable electronic devices has increased dramatically. However, powering them in a stable manner remains a challenge due to the ongoing energy crisis worldwide [1]. In this aspect, the triboelectric nanogenerator (TENG) is a promising energy harvesting technology owing to its special ability of converting the low-frequency mechanical energy to electrical energy [2]. Basically, the working principle of TENGs depends on the coupling effect of sequential triboelectrification and electrostatic induction, and the fundamental theory lies in Maxwell’s displacement current and change in surface polarization [3,4]. Based on this principle TENGs are able to harvest energy from green and renewable sources such as body motions, ocean weaves and wind flows [5,6,7,8,9]. However, despite the rapid advancement in output performance, the triboelectric surface charge decay and poor stability of tribolayer are some of the critical issues of TENGs and could limit certain practical applications. In these aspects, significant research effort has been devoted to enhancing the surface charge density of tribomaterials such as plasma treatment, surface functionalization of triboelectric materials using corona discharge and micro/nanopatterning of tribosurface area [10,11,12]. Moreover, some researchers are adapting nanostructured surface modification methods, such as templating, appending, etching and crumpling, to achieve a high output performance of TENGs [13,14]. Although the above approaches can be used to fabricate the high-performance TENGs, the complicated fabrication processes and high cost of device design may limit the wide range of practical applications. Hence, there is a pressing need to fill the gap towards potential applications and integrate robust technologies to enhance the output performance of TENGs for future flexible electronic applications.

The triboelectrification process involves charge generation, charge storing and charge decay of triboelectric materials [15]. Therefore, selecting the proper triboelectric materials is the key to improve the output performance of flexible TENGs. In this regard, two-dimensional graphene, a monolayer honeycomb lattice structure of sp^2^-bonded carbon atoms, can be a promising material for TENGs owing to its unique properties such as high electrical conductivity, excellent mechanical flexibility, optical transparency and environmental stability [16,17]. The unavoidable wrinkles and ripples of CVD-grown graphene make it more suitable for high output performance due to the enhancement in surface charge during the triboelectrification process. Many studies have reported high output performance of TENG by utilizing graphene as conducting electrode [18,19]. Kim et al. and Liu et al. have demonstrated a flexible TENG, using graphene as a triboelectric material, but unfortunately, the TENG exhibited a low output performance [20,21]. Furthermore, modifying the surface of graphene by plasma treatment, surface modification, micro/nanopatterning is a viable way to enhance the output performance of Gr-TENGs [10,11,12,22]. However, these methods create defects in the graphene surface, which may deteriorate the graphene and affect device performance. To solve the above problem, many researchers have adopted the interfacial modification of TENG for enhanced output performance [23,24]. Extensive research has been conducted to study the effect of the charge trapping layer on TENG performance. Wu et al. demonstrated a significant enhancement in the surface charge density of TENGs by using reduced graphene oxide (rGO) as a charge trapping layer (CTL) under the friction layer [25]. Furthermore, Cui et al. have shown the improvement in the output performance of TENGs by extending charge decay time and enhancing induced charges with the addition of dielectric layer and charge transport layer in between the triboelectric material and contact electrode [26]. However, most of the previous work has focused on negative triboelectric charge enhancement. Recently, another group has reported high output performance by enhancing the positive charge trap [27]. Based on these previous studies, it has been proved that the enhancement of output performance of TENG presents a positive correlation with the increase in surface charge density and charge trapping sites without degrading the properties of triboelectric material. However, the role played by the charge trapping with multilayer structure has not been investigated. Thus, it is necessary to analyze the multilayer structure with CTL, which influences the output performance of a TENG.

In this work, we report a new approach to achieve high output performance of layer-by-layer assembled multilayer graphene-based TENG by introducing Al_2_O_3_ CTL in between the positive triboelectric layer and the bottom conducting electrode. The Gr-TENG with Al_2_O_3_ introduces a mechanism of surface charge enhancement in conduction domains. Relying on the synergistic effect of optimized graphene layers and high dielectric Al_2_O_3_ CTL, there is a large triboelectric charge yield. The optimized flexible 3L-Gr-TENG with Al_2_O_3_ exhibits an enhanced output voltage and current of ~55 V and 0.78 µA, respectively. These values were nearly 5-fold higher than those of the counterpart pristine 3L-Gr-TENG (without Al_2_O_3_ CTL). The output power of the 3L-Gr-TENG is increased from 0.77 to 25 µW (~30 times higher) with the Al_2_O_3_ CTL under ambient conditions. Importantly, by taking advantage of multilayer graphene as a positive triboelectric layer, the bottom graphene layers can act as a charge transport bridge between Al_2_O_3_ and the top graphene layer due to its high electrical conductivity, thus accumulating more positive charge on the graphene surface and facilitating the electron flow from graphene to the opposite triboelectric layer. Moreover, the device shows high stability and durability after continuous operation of >2000 cycles. Furthermore, the generated power can light up more than 20 commercial green LEDs and charge various capacitors to power an electronic timer through the rectifying circuits. Most importantly, this work demonstrates a novel and cost-effective method to improve the performance of flexible Gr-TENGs, which can be a power candidate for next-generation flexible energy harvesting systems.

## 2. Experimental Section

### Fabrication of a Gr-TENG with Al_2_O_3_ as the CTL

**Figure 1:**
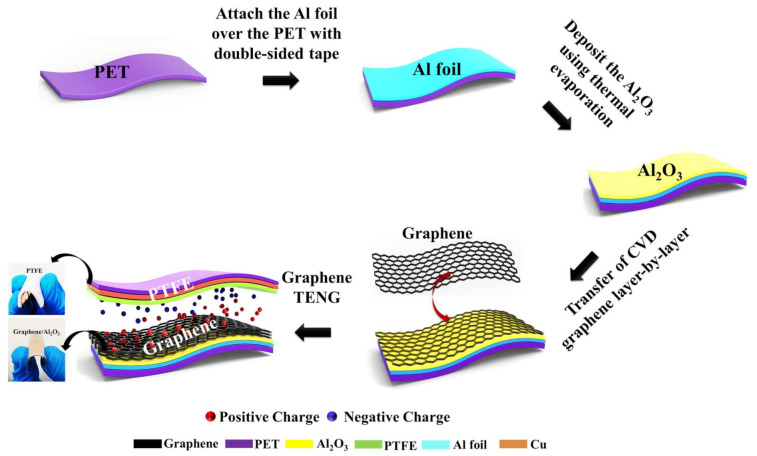
Schematic illustration showing the fabrication process of a flexible Gr-TENG with Al_2_O_3_ as the CTL.

Figure 1 shows the schematic diagram for the fabrication process of a flexible Gr-TENG with Al_2_O_3_ as the CTL over a PET substrate. In this study, the conductor-to-dielectric contact mode was used. To design the vertical contact–separation mode TENG device, the positive friction layer of the TENG was fabricated as follows: (i) First, a 3 × 3 cm^2^, 80 μm thick commercial aluminum (Al) foil was taken as the conducting electrode, which was attached over the polyethylene terephthalate (PET) substrate (~188 μm) with double-sided tape. Then, the Al-foil/PET substrate was cleaned by ethanol and dried in a stream of N_2_ gas. (ii) Thereafter, a thin layer of Al_2_O_3_ (~10 nm), as the CTL, was thermally deposited over the Al-foil/PET substrate by the thermal evaporation method [28]. (iii) Finally, the CVD-grown graphene [29], as a positive friction layer, was transferred over the Al_2_O_3_/Al-foil/PET substrate using the PMMA transfer method, as shown in Figure S1. In this work, we used monolayer (1L) and multilayer (3L and 5L) graphene as the positive friction layer. Multilayer graphene (3L and 5L) was fabricated by transferring monolayer graphene layer by layer over the Al_2_O_3_/Al-foil/PET substrate. The positive charge trapping nature and higher relative permittivity of Al_2_O_3_ helped to enhance the surface charge over graphene for high output performance [30]. In addition, the improvement in the surface roughness of the graphene layer due to Al_2_O_3_ provided extra support for the enhancement in the electrical output of the Gr-TENG. Subsequently, a commercially available polytetrafluoroethylene (PTFE) film (3 × 3 cm^2^) served as the negative friction layer due to its high electronegativity, which could accept more electrons when rubbed against a positive friction layer [31]. A conducting copper electrode was deposited on the back side of the PTFE film using thermal evaporation. It is noted that the PTFE and graphene surface were placed face-to-face, leaving a small gap between the two contact surfaces. Two thin copper wires were connected to the conducting electrodes (copper and Al foil) to form a complete TENG device. The flexibility of graphene and PTFE is clearly shown in the Figure 1. Such fabrication steps clearly demonstrate an easy and cost-effective fabrication process of the proposed Gr-TENG, which can be suitable for wearable electronic devices.

## 3. Results and Discussion

### 3.1. Material Characterization of Graphene Layers/Al_2_O_3_

**Figure 2:**
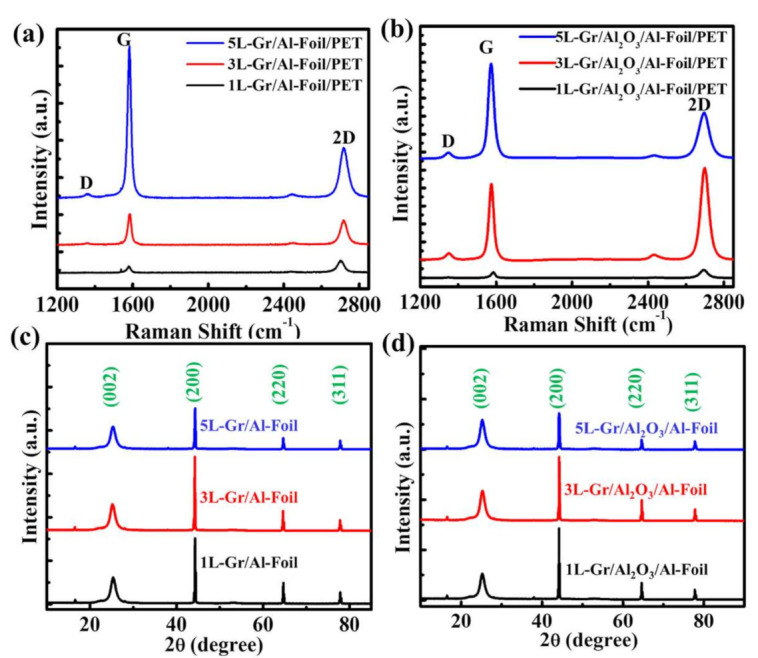
The Raman spectra of (**a**) graphene/Al-foil/PET and (**b**) graphene/Al_2_O_3_/Al-foil/PET. XRD patterns of (**c**) graphene/Al-foil/PET and (**d**) graphene/Al_2_O_3_/Al-foil/PET.

Raman spectroscopy is a powerful and nondestructive tool to analyze the quality of graphene. Figure 2a,b shows the Raman spectra of 1L, 3L and 5L graphene before and after their transfer over the Al_2_O_3_/Al-foil/PET substrate. Figure 2a shows the graphene layers (1L, 3L and 5L) over the Al-foil/PET substrate, where the G peak (at ~1582 cm^−1^) and the 2D peak (at ~2700 cm^−1^) are the characteristics of the sp^2^-hybridized C–C bonds in graphene. Basically, these two bands are used to determine the number of layers in graphene. In addition, a negligible D peak (at ~1359 cm^−1^) corresponds to atomic-scale defects or lattice disorder in graphene [32]. Here, the negligible intensity of the D peak indicates the low density of defects and a highly crystalline phase of graphene. However, with an increase in the number of graphene layers (3L and 5L), the G band becomes more intense, and the 2D peaks become broader and slightly upshifted with respect to monolayer graphene [33], as shown in Figure 2a. The slight upshift may be due to the unintentional strain originating from the growth of the copper substrate and the unavoidable formation of wrinkles during the graphene transfer process [34]. Moreover, the I_2D_/I_G_ of ~2.18 of CVD-grown graphene indicates high-quality monolayer graphene and gradually decreases with an increase in the number of layers, as shown in Figure S2a. The intensity of the D peak slightly increases after transferring the graphene layers (1L, 3L and 5L) over the Al_2_O_3_/Al-foil/PET substrate, as shown in Figure 2b and Figure S2b. This result indicates an increase in substrate roughness due to the deposition of thin-layer Al_2_O_3_ over the Al-foil/PET substrate, which is beneficial for enhancing the output performance because the effective contact increases during the triboelectrification process.

To further confirm the quality of graphene (1L, 3L and 5L) on the Al-foil/PET and Al_2_O_3_/Al-foil/PET substrates, we performed XRD, which is shown in Figure 2c,d. Both figures show that the diffraction peak at a 2θ of 26.4° corresponds to the (002) lattice orientation of hexagonal graphitic carbon, which indicates the successful fabrication of high-quality graphene [35]. Additionally, the other peaks at 44.7, 65.1 and 78.3° correspond to the (200), (220) and (311) lattice orientations, respectively, which are attributed to Al (JCPDS card No. 04-0787) as the graphene is transferred over the Al-foil substrate. It is clearly visible that the positions of different graphene layers over the Al-foil/PET and Al_2_O_3_/Al-foil/PET substrates retain the same peak position, indicating that the crystalline structure of graphene is restored after transfer over the Al_2_O_3_/Al-foil/PET substrates, which is helpful for the enhancement of the electrical output of the TENG.

**Figure 3:**
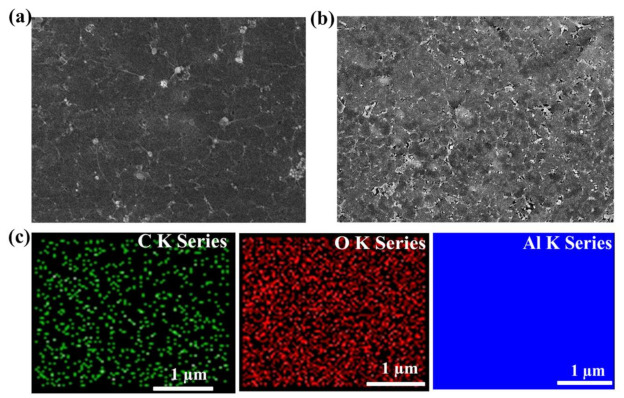
FESEM image of the graphene surface on (**a**) Al-foil/PET and (**b**) Al_2_O_3_/Al-foil/PET. (**c**) EDS elemental mapping of the graphene/Al_2_O_3_/Al-foil/PET presenting C K series, O K series and Al K series.

Further, to investigate the surface morphology of graphene after the insertions of Al_2_O_3_ CTL, FESEM analysis was conducted, as illustrated in Figure 3a,b. Regarding the FESEM analysis, a sample size of 1 cm^2^ was used. As clearly seen from Figure 3b, the graphene over Al_2_O_3_/Al-foil/PET substrate exhibits a rougher wrinkled surface morphology in comparison with the graphene over Al-foil/PET substrate (Figure 3a) due to the underneath nanostructure roughness of Al_2_O_3_, which supports the output enhancement of TENGs due to the enhancement of effective contact area during triboelectrification process. Additionally, we performed energy-dispersive X-ray spectroscopy (EDS) to quantify the atomic composition of the graphene sample. The graphene samples contain carbon (C), oxygen (O) and aluminum (Al), as depicted in Figure S3. Furthermore, the EDS elemental mapping (Figure 3c and Table 1) confirms the uniform distribution of C, O and Al. According to the elemental analysis, the occurrence of aluminum (44.51%) and the low atomic percentage of oxygen (1.10%) are due to the transfer of graphene over conducting electrode (Al foil) and the unavoidable oxidation of graphene. However, the atomic percentage of oxygen gradually increases to 6.08 % (Table 1) when the graphene is transferred on the Al_2_O_3_/Al-foil/PET substrate. This increase in oxygen will help to enhance the output performance because oxygen has excellent electron-donating ability due to its high Lewis basicity, which makes the graphene layer more tribopositive [36].

**Figure 4:**
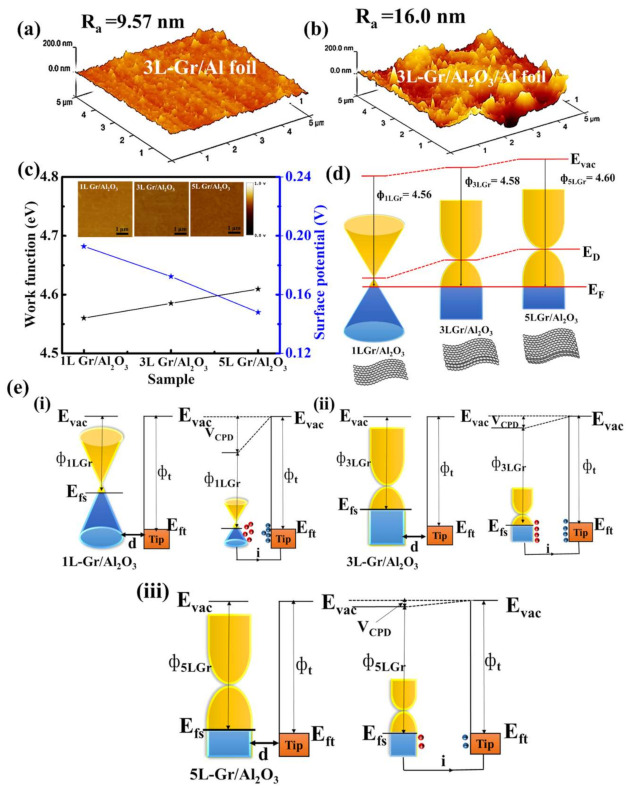
3D AFM images of (**a**) 3L-Gr/Al-foil/PET substrate and (**b**) 3L-Gr/Al_2_O_3_/Al-foil/PET substrate. (**c**) Work function measurements of 1L-, 3L- and 5L-Gr on the Al_2_O_3_/Al-foil substrate by KPFM. Inset showing the surface potential of graphene layers (1L, 3L and 5L) over Al_2_O_3_. Schematic illustration of (**d**) energy band diagrams for 1L-Gr, 3L-Gr and 5L-Gr over Al_2_O_3_. (**e**) Electronic energy levels of graphene samples and AFM tip without and with electrical contact for three cases: (**i**) tip and the 1L-Gr over Al_2_O_3_/Al-foil/PET, (**ii**) tip and the 3L-Gr over Al_2_O_3_/Al-foil/PET and (**iii**) tip and the 5L-Gr over Al_2_O_3_/Al-foil/PET. E_vac_ is the vacuum energy level. E_fs_ and E_ft_ are Fermi energy levels of the graphene sample and tip, respectively. V_CPD_ is the contact potential difference, and Φ_nGr_ and ϕ_t_ are work functions of the graphene layer (n = 1, 3 and 5) and tip, respectively.

Typically, the surface roughness of triboelectric materials plays a crucial role in the enhancement of TENG output performance. Thus, to examine the surface roughness of the triboelectric layer, we performed AFM analysis. Figure 4a,b shows the 3D AFM image of the three-layer graphene on the Al-foil/PET and Al_2_O_3_/Al-foil/PET substrates. The surface roughness values of graphene (1L, 3L and 5L) on the Al-foil/PET substrate were 7.78, 9.57 and 11.2 nm, respectively (Figure S4a,b). However, the surface roughness of graphene (1L, 3L and 5L) was further enhanced to 11.8, 16.0 and 14.2 nm after the fusion of Al_2_O_3_ CTL underneath the graphene layers (Figure S4c,d). This result indicates that the random layer-by-layer transfer of graphene and the nanostructure surface roughness of Al_2_O_3_ enhance the surface roughness of graphene layers [37]. However, after three layers of graphene transfer, the subsequent layers are not much more affected by the surface roughness of Al_2_O_3_ due to the increase in thickness of the multiple layers of graphene. Therefore, the surface roughness of 5L-Gr is less than that of 3L-Gr over the Al_2_O_3_/Al-foil/PET substrate. Regarding TENGs, the increase in the surface roughness of graphene is beneficial for enhancing the electrical output because the surface roughness increases the effective contact area of the graphene friction layer. Regarding TENGs, the increase in the surface roughness of graphene is beneficial for enhancing the electrical output because the surface roughness increases the effective contact area of the graphene friction layer.

In addition, we have investigated the work function of graphene layers using KPFM to comprehensively understand the physical mechanism of triboelectric graphene layers on the output performance of TENGs. Figure 4c shows the work function (WF) and surface potential of the graphene layer (1L, 3L and 5L) on the Al_2_O_3_/Al-foil/PET substrate. The corresponding work functions were measured to be Φ_1LGr_ = 4.56 eV, Φ_3LGr_ = 4.58 eV and Φ_5LG_ = 4.60 eV, respectively. The measured WF of graphene follows an increasing trend with an increase in the number of layers, which agrees well with the previously reported literature [20,38]. A similar trend occurs when graphene is transferred on the Al-foil/PET substrate as shown in Figure S5. However, the surface potential of graphene gradually decreases with an increase in the number of graphene layers (see the detail in Figure S6). The key factors for the gradual increase in the WF of graphene can be explained as follows: (i) the shift in the Fermi level of graphene with respect to the number of layers (Figure 4d) affects the charge transfer at the substrate interface, and the charge distribution within the graphene layers affects the WF of graphene; (ii) the increase in graphene thickness with respect to the number of layers may affect the WF of graphene; and (iii) the underlying substrate is also an important factor that affects the WF of graphene [39]. Moreover, the change in WF affects the surface potential of graphene layers [40], as illustrated in Figure 4e. When the 1L-Gr/Al_2_O_3_ sample and tip are brought close to each other for electron tunneling, the equilibrium forces align the fermi level of the 1L-Gr sample with the tip. Upon electrical contact, the 1L-Gr/Al_2_O_3_ sample and tip are charged, and V_CPD_ is formed between the tip and 1L-Gr/Al_2_O_3_ sample (Figure 4e(i)). With the increase in the number of layers (3L and 5L), the characteristic energy level of graphene surface further drops, resulting in a smaller contact potential difference, as shown in Figure 4e(ii–iii). Therefore, the proper optimization of the graphene layer is very important for enhancing the output performance of TENGs because the larger potential difference between the triboelectric layer leads to a better output performance.

### 3.2. Working Mechanism of Gr-TENG with Al_2_O_3_ as CTL

**Figure 5:**
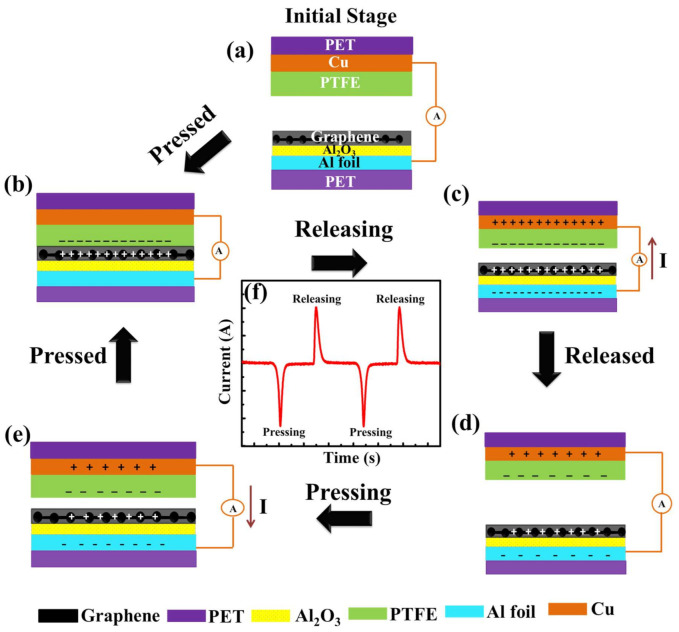
Schematic diagram showing the working mechanism of Gr-TENG with Al_2_O_3_. (**a**) Initial stage of two different triboelectric layers when no external impact is applied. (**b**) Triboelectric charges are generated on the surfaces of graphene and PTFE when they are in contact with each other. (**c**) Separation of graphene and the PTFE triboelectric layer begins. The current flows from the bottom electrode (graphene) to the top electrode (PTFE) to maintain electrical equilibrium. (**d**) Complete separation of the graphene and PTFE triboelectric layer and reaching electrical equilibrium causes no electron flow. (**e**) Pressing the graphene and PTFE triboelectric layer into contact again causes the current to flow from the top electrode to the bottom electrode. (**f**) One typical signal of Gr-TENG with Al_2_O_3_ upon pressing and releasing.

Prior to performing the electrical measurement and examining the relationship between the output performance of the flexible pristine Gr-TENG and Gr-TENG with Al_2_O_3_, the basic operating mechanism of the flexible Gr-TENG with Al_2_O_3_ is clearly elaborated and illustrated in Figure 5. In general, the working mechanism of a TENG is based on the coupling effect, triboelectrification and electrostatic induction effect between triboelectric layers. In the initial position when the triboelectric graphene layers (as the tribopositive bottom layer) and PTFE (as the tribonegative top layer) are separated by a certain distance, there is no charge generation on the surface of graphene and PTFE. Therefore, no electric potential between the electrodes is observed, and no signal is observed (Figure 5a). Once the external impact is applied to the electrode, both triboelectric layers (graphene and PTFE) are brought into contact with each other. According to the triboelectric series, PTFE is much more triboelectrically negative than the graphene layer. Hence, due to the triboelectrification phenomenon, electrons (negative charges) are injected into the PTFE film from the graphene layer while leaving positive charges on its surface, as shown in Figure 5b. Since the high-k dielectric and positive charge trapping nature of Al_2_O_3_ [28] as the CTL exists under the graphene layer, the positive charge density over the graphene surface will be enhanced. This increases the flow of free electrons from the graphene surface to the PTFE film, causing the accumulation of more positive and negative surface charges on the graphene and PTFE surfaces, respectively. When the external force is withdrawn, both triboelectric layers separate, resulting in a potential difference between the electrodes. The difference in electric potential leads to current flow from the positively charged graphene to negatively charged PTFE via an external load (Figure 5c). This current flow is due to the electrostatic induction effect. Afterward, when both triboelectric layers (graphene and PTFE) are completely separated, a new equilibrium state occurs, in which no current conduction takes place, as shown in Figure 5d. After complete separation, if the device is pressed again, a reverse current will flow back through the external load, as depicted in Figure 5e. The repetition of this working mechanism leads to the generation of a periodic alternating current (AC). One typical signal of Gr-TENG with Al_2_O_3_ upon pressing and releasing is shown in Figure 5f. It is important to note that the charge generation and accumulation over the triboelectric layer are strongly related to the optimized graphene layers with the Al_2_O_3_ CTL. Moreover, the tribopositive charge is not only generated on the top surface of multilayer graphene but also generated on the surface of Al_2_O_3_, which continuously supplies the positive charge to the top graphene layers, which will increase the potential difference when the device is released. The high dielectric constant of Al_2_O_3_ CTL enhanced the total capacitance of Gr-TENG, which will support the output enhancement. By considering the contact–separation mode, when the two triboelectric layers contact each other, the open-circuit voltage (V_OC_) at zero transferred charge can be expressed according to the following equation [41]:

(1) $$V_{OC}=\frac{\sigma_{0}\times X\left(t\right)}{\varepsilon_{0}}$$

where $\varepsilon_{0}$, $\sigma_{0}$ and *x*(*t*) represent the vacuum permittivity, surface charge density and distance between graphene and PTFE triboelectric layers, respectively. According to Equation (1), the *V_OC_* is strongly related to the $\sigma_{0}$. However, the surface $\sigma_{0}$ depends on the device capacitance because the TENG acts as both energy storage and energy generation device. Therefore, the device capacitance can be calculated as follows [42]:

(2) $$C_{max}=\frac{\varepsilon_{0}\varepsilon_{r}A}{d}$$

where $\varepsilon_{r}$ is defined as relative permittivity, *A* is the surface area and *d* is the thickness of the triboelectric layer. According to Equation (2), a thin film with high relative permittivity can exhibit high output performance. Therefore, for the enhanced output performance, the optimized number of graphene layers needs to be investigated.

### 3.3. Electrical Characterization of Gr-TENG with Al_2_O_3_ CTL

**Figure 6:**
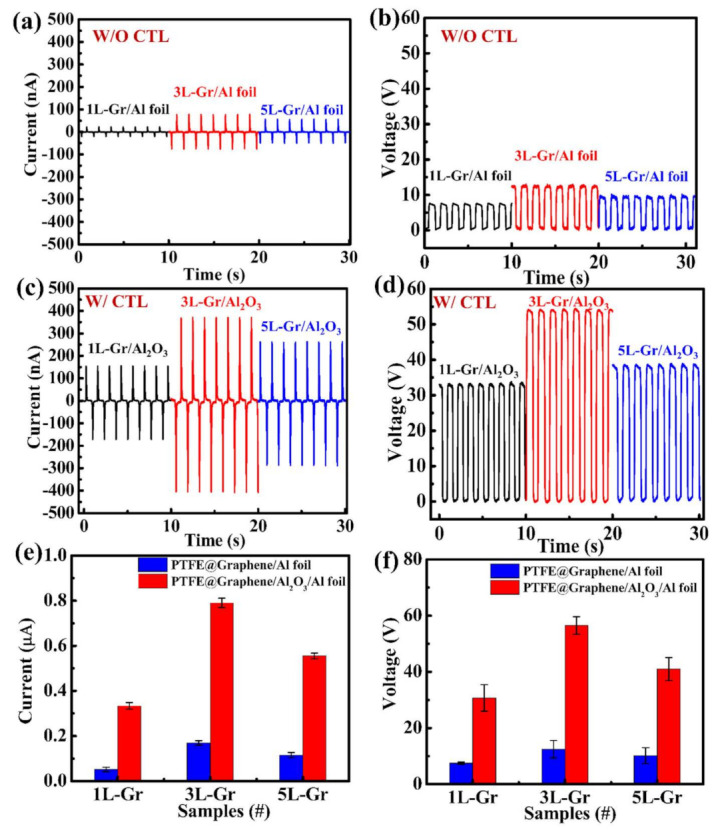
Electrical output of the Gr-TENG: (**a**) Short-circuit current (I_SC_) and (**b**) open-circuit voltage (V_OC_) of 1L-, 3L- and 5L-Gr-TENGs without Al_2_O_3_ CTL. (**c**) I_SC_ and (**d**) V_OC_ of 1L-, 3L- and 5L-Gr-TENGs with Al_2_O_3_ CTL. Average mean (**e**) current and (**f**) voltage generated by pristine Gr-TENGs (1L, 3L and 5L) and Gr-TENGs (1L, 3L and 5L) with Al_2_O_3_ CTL. Error bars indicate standard deviations for 4 sets of data points.

The electrical output current and voltage of the Gr-TENG (1L, 3L, and 5L) with and without Al_2_O_3_ CTL were systematically investigated, as shown in Figure 6. These measurements were performed at a frequency of 1 Hz. A comparison of the electrical output of Gr-TENG (1L, 3L, and 5L) without the Al_2_O_3_ CTL is depicted in Figure 6a,b. Initially, the 3L-GR-TENG over the Al-foil/PET substrate exhibits the maximum I_SC_ of ~155.9 nA and V_OC_ of ~12 V compared to those of 1L-Gr-based (I_SC_ ~43.1 nA and V_OC_ ~7.5 V), 2L-Gr-based (I_SC_ ~66.1 nA and V_OC_ ~9 V (Figure S7)), 4L-Gr-based (I_SC_ ~113.9 nA and V_OC_ ~11 V (Figure S7)) and 5L-Gr-based (I_SC_ ~108 nA and V_OC_ ~9.5 V) TENGs. The experimental results indicate that with the increase in the number of graphene layers, the conductivity gradually increases due to the decrease in sheet resistance, as shown in Figure S8, which enhances the output I_SC_ and V_OC_ of the 3L-Gr-TENG. However, there is a decrease in electrical output for the 4L- and 5L-Gr-based TENGs compared with the 3L-Gr-based TENG. It should be noted that the increase in output I_SC_ and V_OC_ not only depends on the conductivity but also has a relationship with the work function of the triboelectric layer (i.e., the graphene in this work). The work function of graphene increases with an increase in the number of graphene layers, as discussed in Figure 4d. A higher work function means a large amount of energy is needed to extract electrons from the surface. Therefore, there should be a balance between the conductivity and work function to identify the number of graphene layers (here, 3L-Gr) that effectively enhances the output performance of the Gr-TENG. Although the performance of the 3L-Gr-based TENG is better than that of the 1L-, 2L-, 4L- and 5L-Gr-based TENGs, the electrical output of the Gr-TENG is not sufficient for many practical applications.

Furthermore, we anticipate that applying a CTL (Al_2_O_3_) under the graphene will be an effective method to further enhance the output performance of Gr-TENGs because the electrical output of TENG strongly depends on the surface charge density [42]. Therefore, following the above experimental demonstration of Gr-TENG with respect to the number of graphene layers as a proof of concept, Gr-TENG with Al_2_O_3_ as the CTL structure was systematically investigated. Apparently, the output I_SC_ and V_OC_ of the Gr-TENG with Al_2_O_3_ as the CTL are remarkably enhanced compared with those of the pristine Gr-TENG, as shown in Figure 6c,d. The 1L-, 2L-, 3L-, 4L- and 5L-Gr-TENGs with Al_2_O_3_ possess output I_SC_ and V_OC_ of 0.33 μA and 32 V, 0.43 μA and 37 V (Figure S9), 0.78 μA and 55 V, 0.63 μA and 45 V (Figure S9) and 0.55 μA and 40.3 V, respectively. The enhancement in the output I_SC_ and V_OC_ is mainly attributed to the following: (i) The increase in surface charge density over the graphene surface is due to the high charge storage capacity of Al_2_O_3_ [43]. (ii) The small intrinsic carrier density of Al_2_O_3_ compared with graphene is also one of the dominant factors [44]. Thus, more induced electrostatic charges resulting from triboelectrification may provide an enhanced electrical output of Gr-TENGs. (iii) The relative increase in the surface roughness of graphene due to Al_2_O_3_ increases the contact area between PTFE and the graphene layer during the triboelectrification process, providing additional support for the enhancement in output performance compared with the flexible Gr-TENG without CTL. Furthermore, Figure 6e,f clearly and intuitively reveals the strong dependency of output I_SC_ and V_OC_ of Gr-TENG on the Al_2_O_3_ CTL. Although the outputs increase with the CTL and lower work function of triboelectric material, the 3L-Gr-TENG with Al_2_O_3_ exhibits a maximum electrical output compared to the 1L-, 2L-, 4L- and 5L-Gr-TENGs with Al_2_O_3_. This result indicates that not only the work function but also the higher surface roughness of 3L-Gr-TENG with Al_2_O_3_ (Figure 4b) plays a crucial role in the enhancement of output performance of TENG. Moreover, the high conductivity of multilayer graphene further enhances the output performance of TENG compared to the 1L-Gr-based TENG. Therefore, there should be a balance between the work function, conductivity and surface roughness to identify the number of graphene layers that effectively enhances the output performance of the Gr-TENG with Al_2_O_3_ CTL. Based on the above results and discussion, the 3L-Gr-TENG with Al_2_O_3_ as CTL exhibits the optimal output performance; therefore, we considered this sample for subsequent experiments in this study.

**Figure 7:**
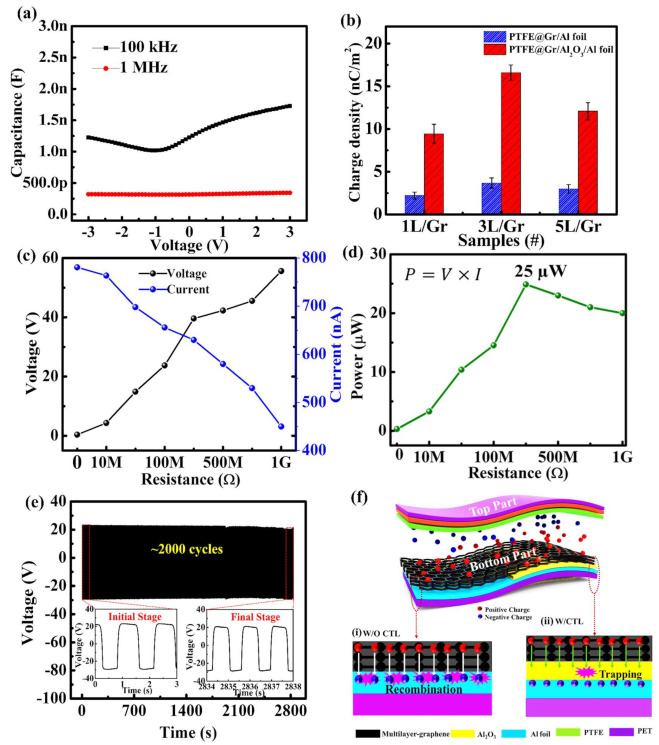
Performances of the 3L-Gr-TENG with the CTL: (**a**) CV characteristics of Al/Al_2_O_3_/3L-Gr/Al at frequencies of 100 kHz and 1 MHz. (**b**) Surface charge density of graphene (1L, 3L and 5L)-based TENG with and without Al_2_O_3_ as CTL. (**c**) Dependence of the output voltage and current outputs as a function of different resistors as external loads. (**d**) Relationship between electrical output power and external loading resistance. (**e**) Mechanical stability and durability test of the TENG with the continuous application of ~2000 cycles. The inset shows the output voltage at the initial stage and the final stage after ~2000 cycles. (**f**) Schematic illustrations showing the charge-trapping mechanism of 3L-Gr-TENG without and with Al_2_O_3_ charge trapping layer.

To experimentally investigate the effect of the charge trapping layer in Gr-TENGs, we fabricated a metal–insulator–metal (MIM) device structure in which 3L graphene over Al_2_O_3_ was sandwiched between the metal electrodes. CV analysis was performed to detect the charge trapping phenomenon. Figure 7a shows the CV curve of the Al/3L-Gr/Al_2_O_3_/Al device at two different frequencies. The capacitance values at 100 kHz and 1 MHz are ~1.25 nF and ~340 pF, respectively. The decrease in capacitance value with increasing frequency is due to the reduced space charge polarization effect [45]. Furthermore, the significant increase in the hysteresis window with increasing sweep voltage, as shown in Figure S10, indicates the increased trapping of charge carriers in the Al_2_O_3_ CTL. This charge trapping capability of graphene over Al_2_O_3_ promotes the triboelectric charge storage and accumulation for the enhanced TENG output performance. Moreover, the surface charge densities (σ) of Gr-TENG (1L-, 3L- and 5L-Gr) with Al_2_O_3_ are comparatively higher than that of Gr-TENG without CTL, as shown in Figure 7b. This indicates that surface charge density is influenced by not only the number of layers but also the existence of Al_2_O_3_ CTL. However, with the increase in the number of layers (from 1L-Gr to 3L-Gr) with Al_2_O_3_ CTL, the surface charge density increases, but the further increase in the number of layers (5L-Gr) does not contribute to the increase in surface charge density of Gr-TENG, which supports the electrical analysis as demonstrated in Figure 6e,f. For practical applications, Figure 7c shows the effect of the output voltage and current of the optimized flexible 3L-Gr-TENG with Al_2_O_3_ as a function of external load resistance ranging from 10 MΩ to 1 GΩ. The TENG output voltage gradually increases with increasing load resistance, while the output current value follows the opposite trend due to ohmic loss. Figure 7d shows the effective electrical output power of the graphene TENG with Al_2_O_3_ as charge trapping layer as a function of external load resistance. This output power was calculated by using Equation (3):

(3) P = V×I

where *V* and *I* correspond to the output peak voltage and current value at various load resistances. The maximum value of the output power reaches 25 μW at a loading resistance of 300 MΩ, which is 30 times larger than that of the pristine 3L-Gr-TENG, as shown in Figure S11a,b. In addition, the 3L-Gr-TENG with Al_2_O_3_ device shows a maximum power density of 6.25 μW/cm^2^ at a load resistance of ~300 MΩ, which is higher than that of a previously reported graphene-based TENG [20,43,46]. This indicates the high potential of the proposed device to support portable electronic devices.

To further explore the stability and durability of the flexible 3L-Gr-TENG with Al_2_O_3_ as the CTL, we continuously applied >2000 cycles (Figure 7e). Notably, there are no significant changes in the output voltage in the initial and final stages after ~2000 cycles, as shown in the Figure 7e inset, confirming the high stability and durability of the TENG device. This demonstrates the outstanding mechanical stability of graphene material the and continuous supply of positive charge from Al_2_O_3_ CTL to the graphene, which leads to a largely enhanced surface charge density and thus the ability to harvest mechanical energy for a long period of time. Furthermore, Figure 7f shows a schematic illustration of the charge trapping mechanism of the 3L-Gr-TENG with Al_2_O_3_. In the case of pristine 3L-Gr-TENG (Figure 7f(i)), under external impact, when both the triboelectric layers, PTFE (top layer) and 3L graphene (bottom layer), come into contact, triboelectric charges (positive charge over graphene and negative charge over PTFE) are generated on their surfaces according to the triboelectric series, as discussed above. The surface charges can be shifted by the electric field and combined with the induced opposite charges. This charge combination can result in a sharp deterioration in electrical output [47], as observed in Figure 6a,b. In contrast, the insertion of the Al_2_O_3_ CTL in between the triboelectric material (3L-Gr) and conducting electrode (Al-foil) contributes to an enhancement in the surface charge density. The remarkable key mechanism for this enhancement can be explained as follows: (i) The high positive charge storage capacity of high-k Al_2_O_3_ [42] underneath the graphene layers promotes charge retention at the surface, resulting in an enhancement in surface charge density that enhances the output performance of TENG. (ii) The multilayered graphene (3L-Gr) plays the dual role of the triboelectric layer and the charge transport layer between the Al_2_O_3_ CTL and top graphene layer due to its high electrical conductivity, which helps to improve the surface charge density (Figure 7b) since it facilitates the charge accumulation process [26]. (iii) Last but not least, the nanomorphology structure of Al_2_O_3_ CTL underneath the graphene plays an important role in the enhancement of surface charge density due to its enlarged effective contact area compared to the flat surface of pristine 3L-Gr-TENG, which in turn enhances the output performance of the Gr-TENG device. Consequently, all the above demonstrations indicate that the proposed optimized flexible 3L-Gr-TENG with Al_2_O_3_ as the CTL possesses promising practical applications for portable electronic devices.

### 3.4. Applications of the Gr-TENG with Al_2_O_3_ as CTL

**Figure 8:**
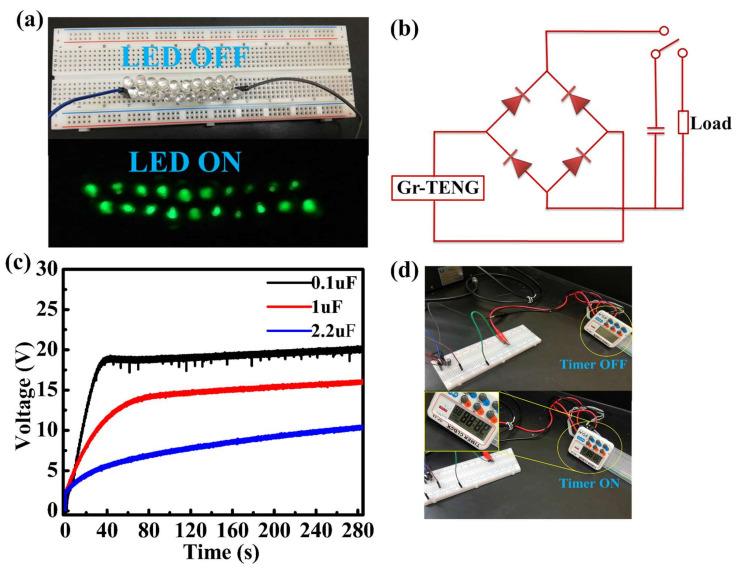
Applications of the Gr-TENG with Al_2_O_3_ as the CTL as a power supply: (**a**) Photograph showing 20 green light-emitting diodes (LEDs) being powered. (**b**) Circuit diagram of the bridge-rectifier for charging a capacitor and turning on a timer. (**c**) Charging curves of capacitors with various capacitances (0.1, 1 and 2.2 µF). (**d**) Photograph of powering a timer.

To further demonstrate the application of a Gr-TENG with Al_2_O_3_ as the CTL as a power source for portable electronics, we successfully lit 20 commercial green light-emitting diodes (LEDs), as shown in Figure 8a (Video S1 in the Supplementary Materials). However, the electrical output of the TENGs is an alternating current (AC) signal that is not suitable to operate portable electronic devices. Therefore, to supply a continuous current to electronic devices, we used a full-wave bridge rectifier circuit to convert the AC signal into a direct current (DC) signal, which was further utilized to charge a commercial capacitor. Finally, the stored energy in the capacitor can be used to operate the electronic device, such as a portable timer. Figure 8b shows the schematic diagram of the bridge rectifier circuit to charge the capacitor in which the capacitor is serially connected to the circuit for energy storage. Figure 8c shows the charging of 0.1, 1 and 2.2 µF capacitors. The capacitor with low capacitance, i.e., the 0.1 µF capacitor, was charged to 19 V in 60 s with the continuous contact–separation process. However, capacitors with high capacitance, namely the 1 and 2.2 µF capacitors, were charged only to 13.6 and 6.3 V in 60 s, respectively. Furthermore, an electronic timer was directly powered by the stored charge, as shown in Figure 8d (Figure S12 and Video S2 in the Supplementary Materials). Although the harvested power may seem low, charging the capacitor allows for the timer to be turned on and requires no battery. Thus, one could envision that by increasing the working area of the Gr-TENG with Al_2_O_3_, it can be used to drive the electronic timer for a long time.

According to the aforementioned experimental results, we hereby conclude that the optimized structure, proper selection of material and surface modification of the tribolayer without degrading its inherent property such as the addition of a CTL to Gr-TENGs promotes the ability to maintain a high surface charge density, resulting in enhanced output power. A brief comparison of the electrical output performance of Gr-based TENGs with and without Al_2_O_3_ CTL is summarized in Table S1. It can be seen that the Gr-TENG with Al_2_O_3_ as CTL shows higher output performance than the Gr-TENG without CTL. These results indicate that the CTL in between the friction layer and conducting electrode is an effective path to improve the triboelectric property of Gr-TENGs. Thus, it is an effective strategy to fabricate the high-performance flexible Gr-TENG with Al_2_O_3_ CTL as an energy harvester.

## 4. Conclusions

In summary, we demonstrated a novel and simple fabrication methodology for enhanced triboelectric performance by introducing Al_2_O_3_ as a CTL between a positive triboelectric material (graphene) and a bottom contact electrode (Al foil). The strong tendency to repel electrons and the positive charge trapping nature of Al_2_O_3_ help to enhance the charge density on the graphene layer. By varying the number of graphene layers (1L, 3L, and 5L) and evaluating the electrical performance, we found the optimal layered structure (3L-Gr) of a flexible Gr-TENG. Maximum V_OC_ and I_SC_ values of ~55 V and 0.78 µA were achieved by the 3L-Gr-TENG with an Al_2_O_3_ CTL. Additionally, this TENG exhibited a maximum power of ~25 μW at a load resistance of ~300 MΩ, which was 30 times higher than that of the pristine 3L-Gr-TENG. Finally, the generated output power was capable of driving 20 commercially available green LEDs connected in series and able to turn on an electronic timer by using a rectifier circuit. Therefore, based on the above results, we believe that our proposed structure holds high promise for enhancing the surface charge density of Gr-TENGs by fusion of CTL, which possesses promising applications for future flexible and portable energy harvesting systems.

## Figures and Tables

**Table 1:** EDS elemental analysis of graphene over Al-foil/PET and Al_2_O_3_/Al-foil/PET.

Elements		Samples	
		Graphene/Al-foil	Graphene/Al_2_O_3_/Al-foil
C K	Weight %	34.90	39.39
	Atomic %	54.39	57.88
O K	Weight %	0.94	5.51
	Atomic %	1.10	6.08
Al K	Weight %	64.16	55.10
	Atomic %	44.51	36.04

## Data Availability

Data are contained within the article or Supplementary Material.

## Supplementary Materials

The following are available online at https://www.mdpi.com/article/10.3390/nano11092276/s1, Materials and method, Figure S1: Schematic illustration of graphene transfers over Al_2_O_3_/Al-foil/PET substrate by PMMA technique, Figure S2: Raman analysis the I_2D_/I_G_ of graphene layers (1L, 3L and 5L) over Al-foil/PET substrate and I_D_/I_G_ of graphene layers (1L, 3L and 5L) over Al_2_O_3_/ Al-foil/PET substrate, Figure S3: EDS spectra of graphene over Al-foil/PET and Al_2_O_3_/Al-foil/PET substrate, Figure S4: 3D AFM images of the transferred graphene layers (1L and 5L) on the Al-foil/PET and Al_2_O_3_/Al-foil/PET substrate, Figure S5: Work function measurements of graphene (1L, 3L and 5L) on the Al-foil substrate, Figure S6: KPFM surface potential measurement of graphene layers (1L, 3L and 5L) over Al-foil/PET and Al_2_O_3_/Al-foil/PET substrate, Figure S7. Electrical output of the Gr-TENG:(a) Short-circuit current (I_SC_) and (b) open-circuit voltage (V_OC_) of 2L- and 4L-Gr-TENGs without Al_2_O_3_ CTL, Figure S8: Sheet resistance of graphene layers (1L, 3L and 5L), Figure S9. Electrical output of the Gr-TENG:(a) Short-circuit current (I_SC_) and (b) open-circuit voltage (V_OC_) of 2L- and 4L-Gr-TENGs with Al_2_O_3_ CTL. Figure S10: CV hysteresis characteristics of 3L-Gr-TENG with Al_2_O_3_ as CTL with different sweeping voltages, Figure S11: Electrical performance of flexible 3L-Gr-TENG without Al_2_O_3_ CTL at various external load resistances, Figure S12: Electrical capacitive load characteristics of Gr-TENG with Al_2_O_3_ as CTL showing the charging and discharging curve for 1 µF capacitor with respect to time; inset shows the powering of a portable electronic timer by the charged capacitor, Table S1. Comparison of electrical output performance of Gr-TENGs with and without Al_2_O_3_ CTL samples used in this study, Video S1: Green LEDs were directly lit up by the Gr-TENG with Al_2_O_3_ CTL, Video S2: An electronic timer was powered using a capacitor charged by the Gr-TENG with CTL.
