# Supplementary material for: Flexible Layered-Graphene Charge Modulation for Highly Stable Triboelectric Nanogenerator
Source: Nanomaterials (Basel). 2021 Sep 1;11(9):2276. doi: 10.3390/nano11092276 (PMC8471573; doi:10.3390/nano11092276)
Supplement: Supplementary file 1 [file nanomaterials-11-02276-s001.zip › nanomaterials-1360206 - SI-Final.pdf]

Supplementary Materials

Flexible Layered-Graphene Charge Modulation for Highly Stable Triboelectric Nanogenerator

Mamina Sahoo [1], Sz-Nian Lai [2], Jyh-Ming Wu [2,3], Ming-Chung Wu [4], and Chao-Sung Lai [1,5,6,7,*]

[1] Department of Electronic Engineering, Chang Gung University, Guishan District, Taoyuan City 33302, Taiwan; msahoo12@gmail.com (M.S.)
[2] Department of Materials Science and Engineering, National Tsing Hua University, Hsinchu 30010, Taiwan; snlai712@gapp.nthu.edu.tw (S.-N.L.); jmwuyun@gapp.nthu.edu.tw (J.-M.W.)
[3] High Entropy Materials Center, National Tsing Hua University, Hsinchu 30010, Taiwan
[4] Department of Chemical and Materials Engineering, Chang Gung University, Taoyuan City 33302, Taiwan; mingchungwu@mail.cgu.edu.tw (M.-C.W.)
[5] Artificial Intelligence and Green Technology Research Center, Chang Gung University, Guishan District, Taoyuan City 33302, Taiwan
[6] Department of Materials Engineering, Ming Chi University of Technology, Taishan District, New Taipei City 24301, Taiwan
[7] Department of Nephrology, Chang Gung Memorial Hospital, Guishan District, Taoyuan City 33305, Taiwan
* Correspondence: cslai@mail.cgu.edu.tw

Materials and Method

Large-area graphene growth: Large-area graphene synthesis was performed over a copper foil (Alfa Aesar, No. 13382, thickness 25 μm, purity 99.8 %) via the low-pressure chemical vapor deposition (LPCVD) method. In detail, graphene synthesis was performed at a high temperature of ~1000 °C using hydrogen (H_2) and methane (CH_4) as source gases. First, the copper foil was placed in a quartz plate and then loaded inside the furnace tube (TF55030, Lindberg/Blue/M). The furnace was heated to 1000 °C at a pressure of 5 mTorr. Then, a gas composed of H_2/CH_4 (1 sccm/100 sccm) was introduced into the chamber for 20 min at a pressure of 450 mTorr to grow graphene. Finally, the LPCVD system was cooled to room temperature at a rate of ~5 °C/s. The grown graphene was transferred to the desired substrate using the well-known PMMA transfer method.

Fabrication of Al_2O_3 as the CTL: We used a thermal evaporation method for the deposition of Al_2O_3 as the CTL over the Al foil/PET substrate. First, a thin layer of Al metal (~1.5 nm) was deposited over the Al foil/PET substrate at a rate of 0.5 Å/s. Immediately after that, the sample was baked at 90 °C for 15 min and exposed to an ambient atmosphere for at least 12 h to form an Al_2O_3 layer. This deposition and oxidation process was repeated seven times to obtain the target oxide thickness of ~10 nm.

Characterization and electrical measurements: Raman spectroscopy was performed to analyze the CVD-grown graphene lattice structure. Raman analysis was performed using an NT-MDT confocal Raman microscope system with a laser beam wavelength of 473 nm, an excitation laser of 2.62 eV, and a laser spot size of ~0.5 μm. To achieve higher accuracy, the analysis was conducted for 30 s with 30 accumulation times for each measurement. AFM analysis and field-emission scanning electron microscopy (FESEM, JEOL JSM-7500F) were used to investigate the surface morphology of graphene. Kelvin probe force microscopy (KPFM, Dimension-3100 Multimode, Digital Instruments) was performed to precisely determine the work function of the graphene layers (1L, 3L and 5L) over the Al foil/PET and Al2O3/Al foil/PET substrates. X-ray diffraction (XRD; D2, Bruker, Germany) was used over a 2θ range of 5 to 90° using a scan rate of 0.04° min−1. The X-ray source was a Cu-Kα radiation source with a wavelength of 1.54 Å at 10 kV and 30 mA. Electrical output measurements, such as the open-circuit voltage and short-circuit current of the TENGs, were carried out using a Keithley 6514 programmable electrometer and a low-noise voltage preamplifier (Stanford Research

Sys-tem Model SR560). The capacitance of multilayer graphene/Al_2O_3/Al foil/PET substrate was measured by using an Agilent B1500A device parameter analyzer.

Figure S1 shows the conventional PMMA transfer method of CVD-grown graphene to the desired Al_2O_3/Al-Foil/PET substrate. First, the PMMA solution was spin-coated over the graphene/copper foil substrate. After coating, the copper foil was etched by immersing the PMMA/graphene/copper foil in copper etchant ($FeCl_3$) for 30 minutes. Afterward, the floated PMMA/graphene film was transferred to the deionized (DI) water (one time ~20 minutes) to remove the residual copper etchant. The PMMA/graphene film was again transferred to the DI water overnight to rinse away the etchant residue completely. Then, the PMMA/graphene film was transferred over the Al_2O_3/Al-Foil/PET substrate. Subsequently, the PMMA layer over the graphene film was removed by acetone followed by IPA solution. For the transfer of multilayer graphene (3L and 5L), the above process was repeated accordingly.

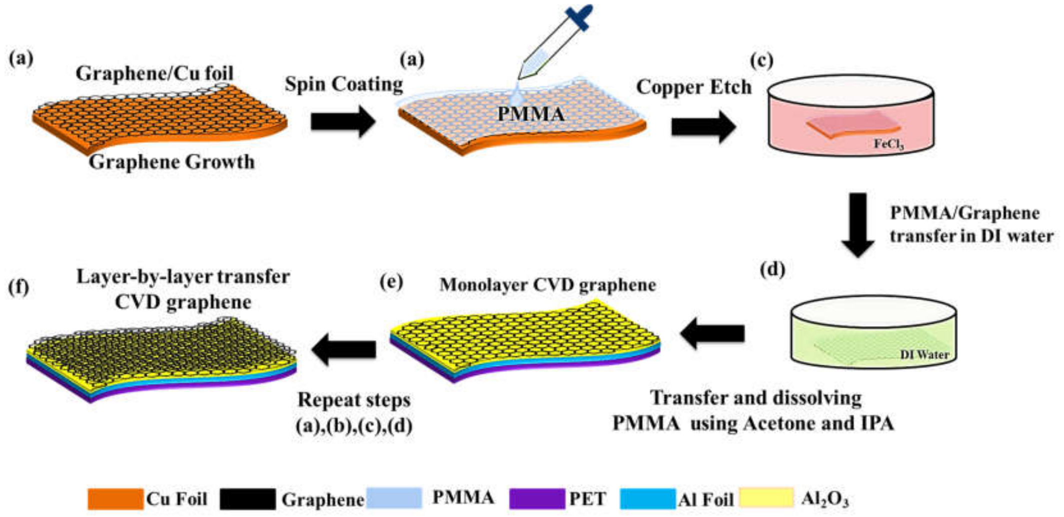

Figure S1. Schematic illustration of graphene transfers over Al_2O_3/Al-foil/PET substrate by PMMA technique.

Figure S2a shows the Raman spectra intensity ratio of 2D-band to G-band (I_{2D}/I_G) of graphene layers (1L, 3L and 5L) over the Al-foil/PET substrate. The I_{2D}/I_G ratio of 1L-Gr was ~2.18 and gradually decreased with the further increase in the number of layers (3L ~0.91 and 5L ~0.58). Similarly, Figure S2b shows that the I_{2D}/I_G ratio of the graphene layers (1L, 3L and 5L) over the Al_2O_3/Al-foil/PET substrate follows the same trends. However, the intensity ratio of D-band to G-band (I_D/I_G) demonstrates the defect density of graphene, which gradually increases with the increase in the number of graphene layers. This indicates the increase in defects density of graphene due to the underlying Al_2O_3 over Al-foil/PET substrate.

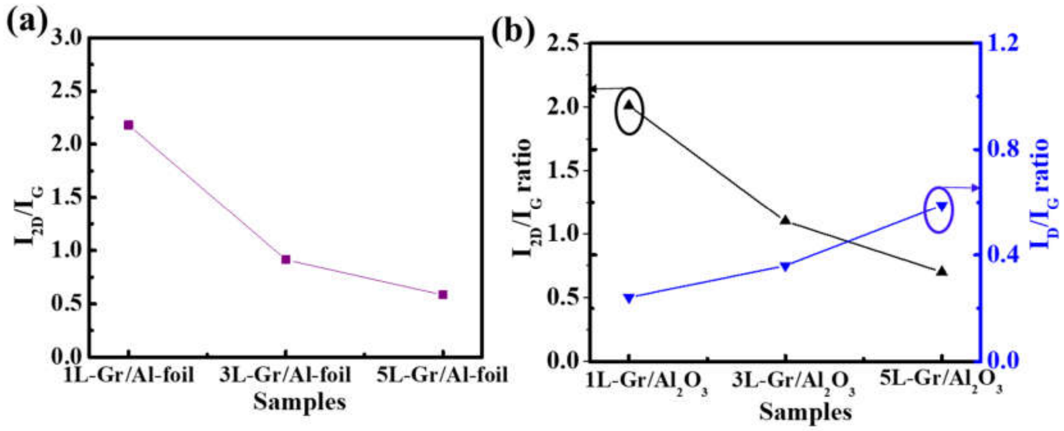

Figure S2. (**a**)The I_{2D}/I_G of graphene layers (1L, 3L and 5L) over Al-foil/PET substrate. (**b**) The I_{2D}/I_G and I_D/I_G of graphene layers (1L, 3L and 5L) over Al_2O_3/Al-foil/PET substrate.

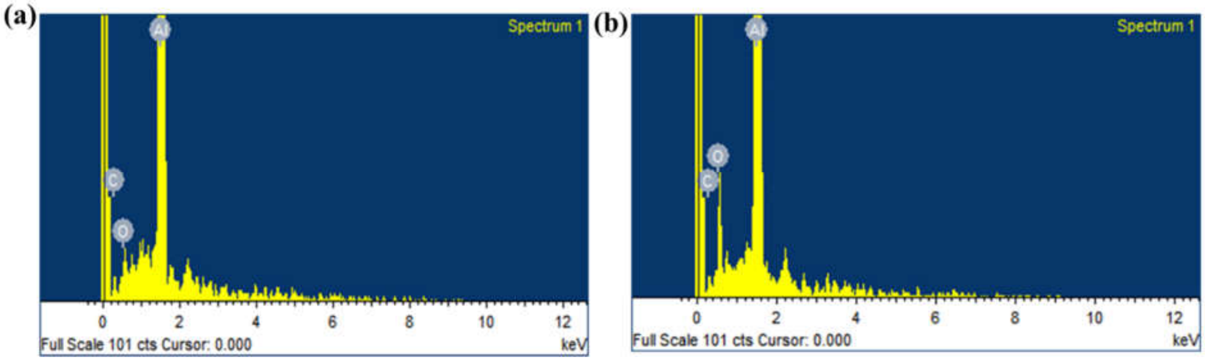

Figure S3. Energy dispersive X-ray (EDS) analysis of (**a**) graphene/Al-foil/PET substrate and (**b**) graphene/Al_2O_3/Al-foil/PET substrate.

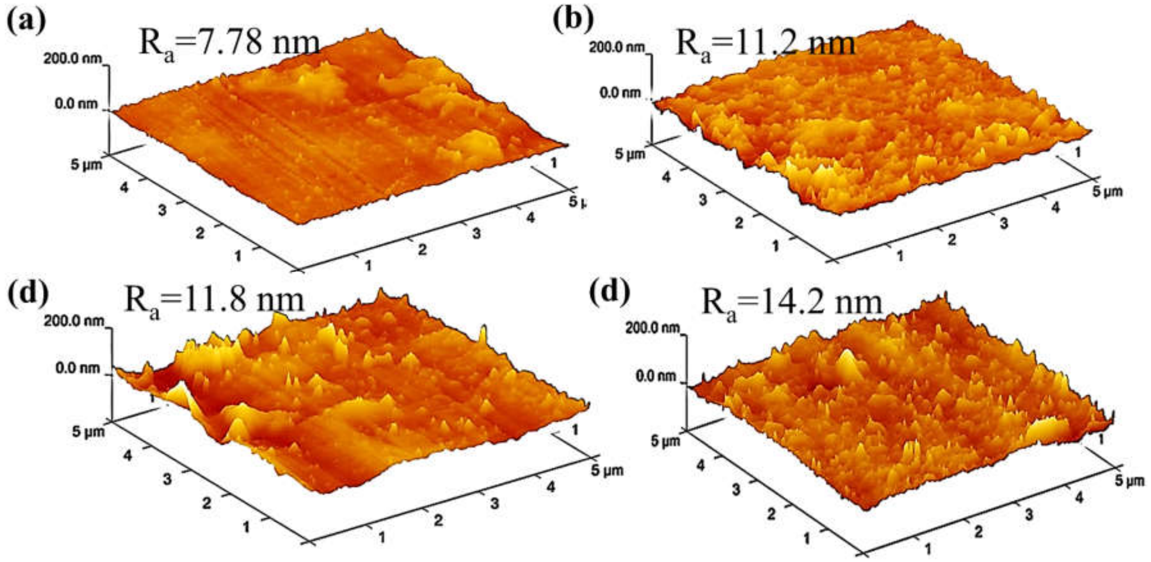

Figure S4. 3D AFM images of the transferred graphene layers: (**a**) single layer (1L) and (**b**) five layers (5L) on the Al-foil/PET substrate. Transferred graphene layers: (**c**) single layer (1L) and (**d**) five layers (5L) on the Al_2O_3/Al-foil/PET substrate.

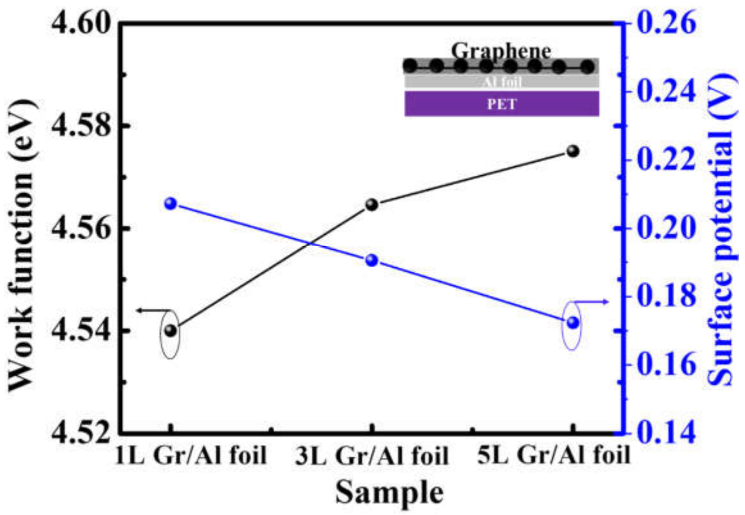

Figure S5. Work function measurements of 1L, 3L and 5L-Gr on the Al-foil substrate.

Figure S6 a, b shows the topography image of the surface potential of graphene layers (1L, 3L and 5L) transferred over Al-foil/PET substrate and Al_2O_3/Al-foil/PET substrate by employing Kelvin probe force microscopy (KPFM). With the increase in the number of graphene layers, surface potential gradually decreases, as shown in Figure S6 a, b.

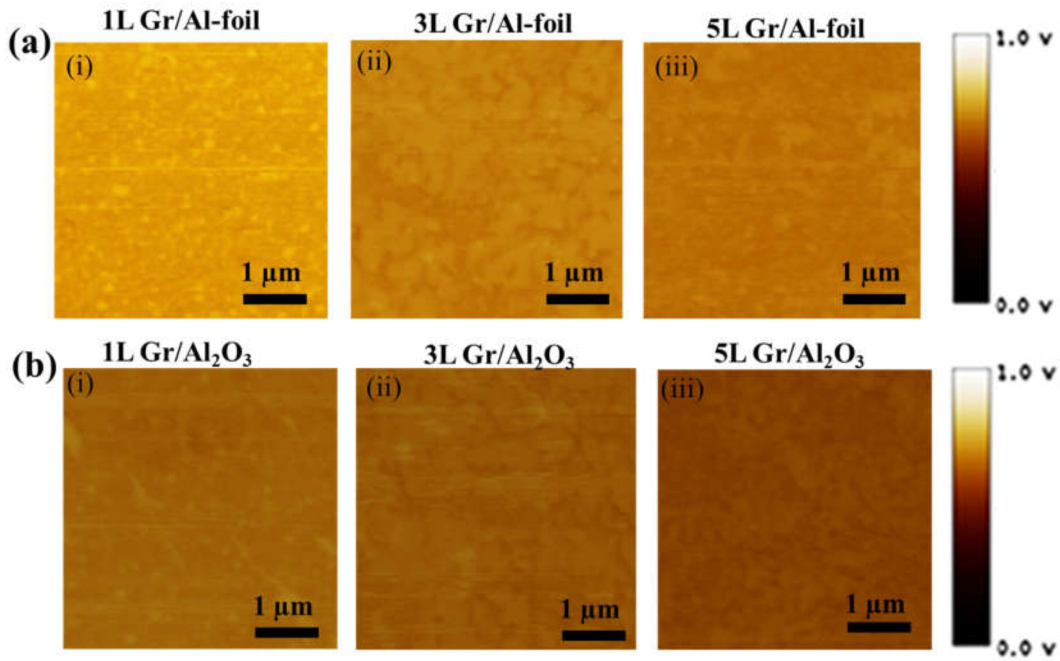

Figure S6. KPFM surface potential measurement data of (**a**) graphene layers over Al-foil/PET ((**i**) 1L-Gr, (**ii**) 3L-Gr and (**iii**) 5L-Gr) and (**b**) graphene layers over Al_2O_3/Al-foil/PET ((**i**) 1L-Gr/Al_2O_3, (**ii**) 3L-Gr/ Al_2O_3 and (**iii**) 5L-Gr/Al_2O_3).

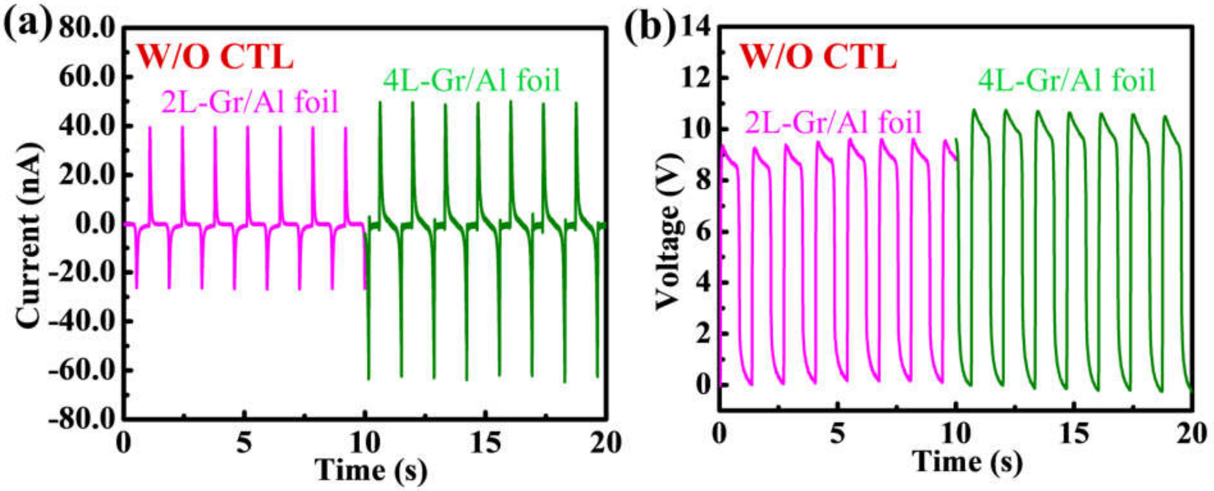

Figure S7. Electrical output of the Gr-TENG: (**a**) Short-circuit current (I_{SC}) and (**b**) open-circuit voltage (V_{OC}) of 2L- and 4L-Gr-TENGs without Al_2O_3 CTL.

Figure S8 shows the sheet resistance of transferred graphene as a function of number of graphene layers. The sheet resistance of a single layer (1L), three layers (3L), and five layers (5L) of graphene formed was about 83 ohm/sq, 31 ohm/sq, and about 22 ohm/sq, respectively. As the sheet resistance decreases with the increase of number of layer indicates the increase in conductivity which will help in the enhancement of the electrical output.

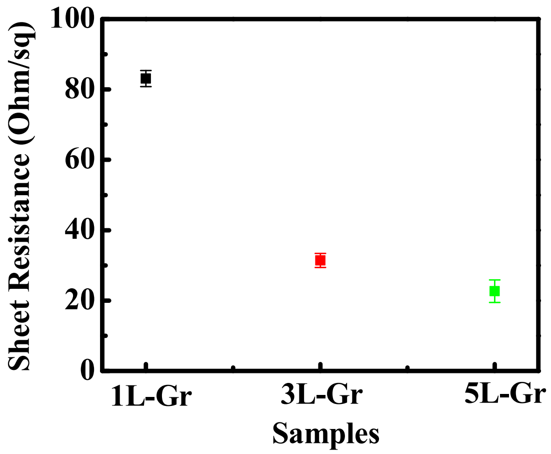

Figure S8. Sheet resistance of graphene as a function of number of layers.

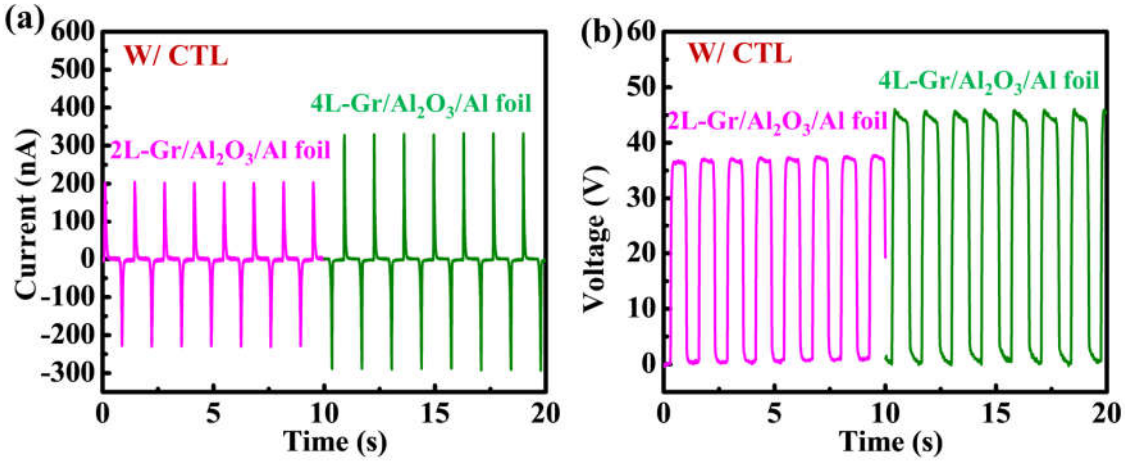

Figure S9. Electrical output of the Gr-TENG: (**a**) Short-circuit current (I_{SC}) and (**b**) open-circuit voltage (V_{OC}) of 2L- and 4L-Gr-TENGs with Al_2O_3 CTL.

Figure S10 shows the measured capacitance of 3L-Gr TENG with Al_2O_3 as CTL with different sweeping voltage. With the increase of sweeping voltage, the hysteresis window getting expands indicates the occupancy of the trapping charge getting increases, which will enhance the surface charge density of Gr-TENG.

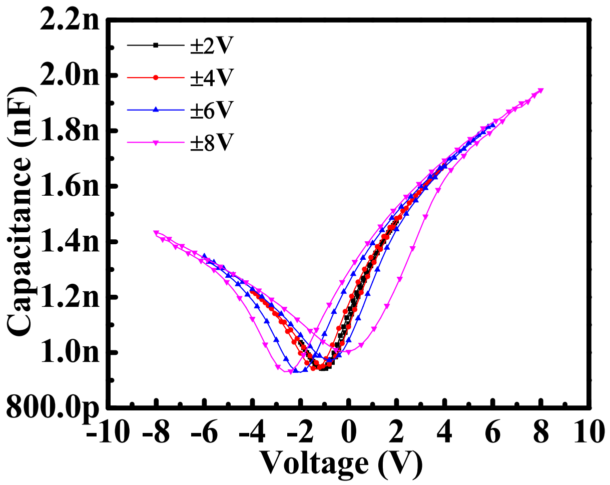

Figure S10. CV hysteresis characteristics of 3L-Gr-TENG with Al_2O_3 as CTL with different sweeping voltages.

Figure S11a illustrates the output voltage and current of pristine 3L-Gr TENG as a function of external load resistance. With the increase of load resistance, the voltage gradually increases whereas the current gradually decreases owing to ohmic loss. The generated peak power of the pristine 3L-Gr TENG was maximized at an external load resistance of 300 MΩ, corresponding to a peak power of 0.77 μW and a power density of 0.19 μW cm^{-2} as shown in Figure S11b.

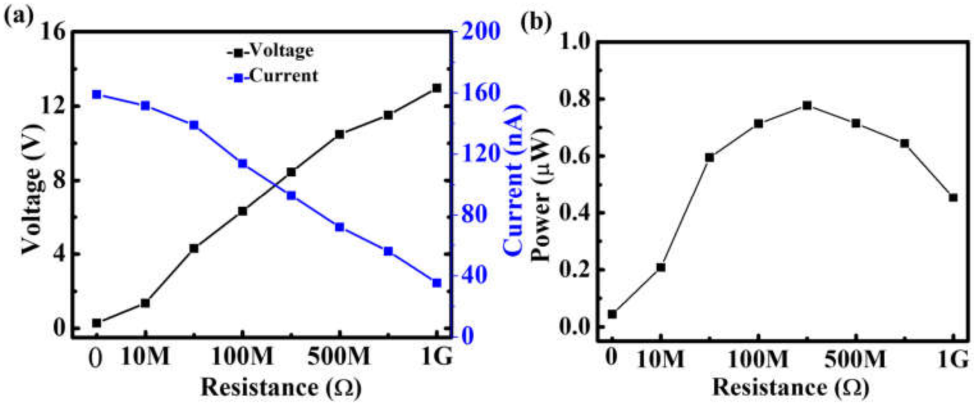

Figure S11. Electrical performance of flexible pristine 3L-Gr TENG (a) Output voltage and current at various external load resistances (b) output power at various external load resistances.

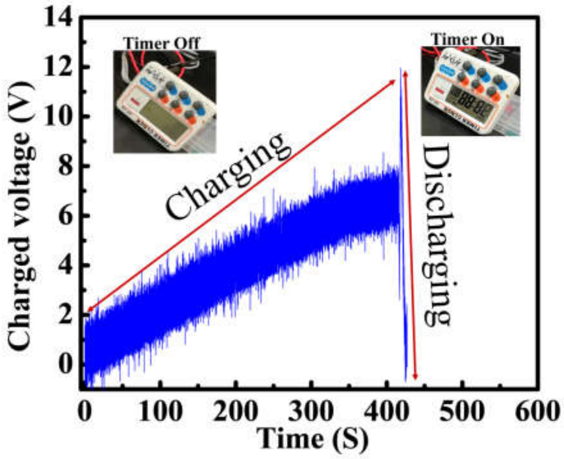

Figure S12. Electrical capacitive load characteristics of Gr-TENG with Al_2O_3 as CTL showing the Charging and discharging curve for (1 μF) capacitor with respect to time and inset shows the powering of a portable electronic timer by the charged capacitor.

Table S1. Comparison of electrical output performance of Gr-TENGs with and without Al_2O_3 CTL samples used in this study.

**Friction Pairs**	**Peak to peak I_{SC}# [nA]**	**Peak to peak V_{OC}# [V]**	**Charge density σ [nC/m²]**
1L-Gr/Al foil/PET@ PTFE	52.1	7.5	2.21
3L-Gr/Al foil/PET@ PTFE	169.225	12.5	3.68
5L-Gr/Al foil/PET@ PTFE	115.3	10.17	3
1L-Gr/Al_2O_3/Al foil/PET@ PTFE	333.5	30.75	9.44
3L-Gr/Al_2O_3/Al foil/PET@ PTFE	790.5	56.5	16.6
5L-Gr/Al_2O_3/Al foil/PET@ PTFE	555.5	41	12.09

#The mean value of I_{SC} and V_{OC} from 4 respective samples.

Supporting Videos

Video S1. Green LEDs were directly lit up by the Gr-TENG with CTL.
Video S2. An electronic timer was powered using a capacitor charged by the Gr-TENG with CTL.

© 2021 by the authors. Licensee MDPI, Basel, Switzerland. This article is an open access article distributed under the terms and conditions of the Creative Commons Attribution (CC BY) license (http://creativecommons.org/licenses/by/4.0/).
